# Systems Medicine Disease: Disease Classification and Scalability Beyond Networks and Boundary Conditions

**DOI:** 10.3389/fbioe.2018.00112

**Published:** 2018-08-07

**Authors:** Richard Berlin, Russell Gruen, James Best

**Affiliations:** ^1^Department of Computer Science, University of Illinois, Urbana, IL, United States; ^2^Department of Surgery, Nanyang Institute of Technology in Health and Medicine, Lee Kong Chian School of Medicine, Nanyang Technological University, Singapore, Singapore; ^3^Lee Kong China School of Medicine, Nanyang Technological University, Singapore, Singapore; ^4^Imperial College, London, United Kingdom

**Keywords:** systems medicine, disease, scalability, networks, boundary conditions

## Abstract

In order to accommodate the forthcoming wealth of health and disease related information, from genome to body sensors to population and the environment, the approach to disease description and definition demands re-examination. Traditional classification methods remain trapped by history; to provide the descriptive features that are required for a comprehensive description of disease, systems science, which realizes dynamic processes, adaptive response, and asynchronous communication channels, must be applied (Wolkenhauer et al., 2013). When Disease is viewed beyond the thresholds of lines and threshold boundaries, disease definition is not only the result of reductionist, mechanistic categories which reluctantly face re-composition. Disease is process and synergy as the characteristics of Systems Biology and Systems Medicine are included. To capture the wealth of information and contribute meaningfully to medical practice and biology research, Disease classification goes beyond a single spatial biologic level or static time assignment to include the interface of Disease process and organism response (Bechtel, 2017a; Green et al., 2017).

## Introduction

The nature of disease and disease states is the conceptual basis of medicine. How patients perceive, how physicians communicate, how education is delivered, how research is forged, all rely on the definition of disease. This construct and the models used to translate and communicate the nature of disease must be adaptable and dynamic, representing contemporary science and models as close to reality as possible; Disease is no longer a stationary term associated with a hierarchy assumed from posterity.

Disease definition is applied in the clinic as well as in the laboratory and thus flexible usage is warranted. Science probes the molecular interior of the cell and the genome and generates interpretations that must satisfy Disease definition (Tillmann et al., 2015; Wang et al., 2015).

Work to map genotype (genetic constitution) to phenotype (observable characteristics) highlights these efforts. (Stadler, 2006; Landry and Rifkin, 2012; Nuzhdin et al., 2012) A New Taxonomy of Disease (taxis—arrangement, nomos—law/science) has been recommended by the National Academy of Science (NAS) to adapt emerging science to clinical medicine and treatment discovery particularly in light of the advances in genome analysis. But there are obstacles to such a New Taxonomy, an enhanced definition of disease (Desmond-Hellman et al., 2011). The International Statistical Classification of Diseases and Related Health Problems (ICD) and its many related derivatives are the standard, universal disease coding method. More than 100 years since its origin, ICD embraces new science and technology but does so through additional codes and alphanumeric place-holders. Once assigned, common usage of ICD creates disease codes which remain static, particularly when placed in patient health system records. From a medical point of view, the continuous description of a clinical course of disease utilizes medical tests characterized by lines, boundaries, and thresholds. But, there remains no facile ICD means to represent these measured changes in patient response to disease; when considering the forthcoming granular tests, including genome studies and molecular analytics (OMICS), such problems only increase. As a result medicalization and over diagnosis persist because medical linear boundaries and thresholds are easily moved or replaced; more alphanumeric codes and deeper testing have not brought science closer to meaningful disease definition that truly represents patient change or biologic depth.

A New Taxonomy that recognizes contemporary science consequently requires the incorporation of systems science: that complex systems, particularly in biology and medicine, are made up of dynamic, adaptive subsystems. These subsystems are managed through competing communication channels and resultant emergent properties. The inclusion of diverse spatial and temporal models is required to enhance definition of a contemplated New Taxonomy.

Systems Biology and Systems Medicine are the means to view Disease as process and response, the interface of inciting agents and continuing organism adaptation (Wolkenhauer et al., 2013; Bechtel, 2017a; Green et al., 2017). A Systems Medicine classification of Disease is the framework for an enhanced definition—giving to Disease itself the participatory characteristics of Systems Biology. Disease is impact and response at the interface between the organism and the environment. Scaling properties must be included and communication pathways defined.

## Disease classification through history

Any addition to disease definition is constrained by historical methodology. Classification methods are fundamental in biology and were applied to botany in the seventeenth century. The result was a framework that related similar characteristics among plants, organizing botany in an eventual tree-like structure of order, descending from broadest general category to most specific example. Classification allows the comparison of common characteristics and subtle differences. However, in the seventeenth century, the emphasis was on listing as many plants as possible and completing individual descriptions for each: to provide order, to provide a label and a reference for comparison and subsequent data analysis (Jutel, 2011; Moriyama et al., 2011; Nordenfelt, 2013).

Sydenham, an English physician influenced by work in the natural sciences, composed the first comprehensive classification for disease published in 1,685 as the Opera Omnia (Anstey, 2011; Sydenham, 2011). Based largely on patient symptoms and viewing individual diseases as unique entities, Sydenham established a compendium, a classification hierarchy modeled on studies in botany which contained disease headings centered on symptoms. Diseases were thought to exist independently in the natural world and their manifestations and clinical expression were completely described as one might a plant.

In the eighteenth century de Sauvages in Paris revisited disease classification and emphasized a patient-symptom-centric structure rather than the disease as entity approach of Sydenham. Keeping to the structure of his predecessor while communicating frequently with the botanist Linneas (the botanist known for the formalized plant classification system: genus, species, etc. that remains in common use today), de Sauvages composed a more formal hierarchical, branching framework. Topic headings clustered disease entries around symptoms (Moriyama et al., 2011).

By the nineteenth century clinical signs of illness from physical examination noted by healthcare providers) were being added to symptoms of disease for a more complete picture; laboratory information and technological advances such as radiography and electrocardiography were also associated with identified disease (Walker, 1990). Leading medical experts at the bedside recognized expressions of disease and developed a robust clinical understanding (Moriyama et al., 2011; Loscalzo et al., 2017).

Bertillon, the Chief of Statistical Services of the City of Paris late in the nineteenth century, was charged with completing a “cause of death” list to track disease, particularly infections, for the city's vital statistics (American Public Health Association, 1899). Bertillon utilized the hierarchical botany-like approach of de Sauvages but re-organized diseases under organ system chapter headings instead of the previous symptom-based format. This remains the classification structure in common use today and even determines the presentation within medical texts and educational courses (Cassell and Siegler, 1985; Nordenfelt, 2013; Vale and Cardoso, 2015).

Bertillon's classification based on the organ system was recognized in the United States in 1898 and subsequently by many national governments; it was officially adopted by the World Health Organization when it came into being after 1946. Called The International Statistical Classification of Diseases and Related Health Problems (ICD), it has become the international standard for disease and health documentation from which originate many derivative classifications^1^. Revised periodically to include new knowledge and disease determinant factors, the current ICD-10 is a tree-like hierarchical alphanumeric coding system with more than 60,000 disease codes each of which contains up to seven alphanumeric place-holders (Jutel, 2011; Moriyama et al., 2011).

ICD captures vital health statistics well and is used in most healthcare systems to encode electronic patient records; but ICD does little to aid the clinical needs of physicians and providers at the bedside or the depth of study required by researchers in the laboratory (Malterud and Hollnagel, 1997). Patient status change or precise representation of clinical disease is poorly supported through ICD encoded records; research must rely on the assignment of ICD and derivative disease associated labels to relate bench investigation with suspected disease conditions. In the case of genome research, this often means associating genotype discovery with ICD patient records for disease assignments (Campbell et al., 1979; Desmond-Hellman et al., 2011; Jutel, 2011; Weiskopf and Weng, 2013). ICD determinations are made using linear boundaries and thresholds in clinical and investigational assessment on the one hand and definitional boundaries on the other. Thus, ICD classifications and codes are static once assigned, very much like disease names themselves. They do not incorporate change, further characterization of disease process or emergent properties.

## Medicalization and over diagnosis—problem of disease definition and fixed boundaries

Medicalization is the assignment of a medical term to a condition where medical science does not provide a basis for classification as a disease; there are usually cultural and social forces that encourage a medical view of such a condition rather than relying on firm science and diagnostic progress (Sholl, 2017).

Over diagnosis occurs when a disease label is used for a condition which might never cause symptoms or harm a patient. This may happen through the interpretation of medical tests or patient symptoms in the face of marginal evidence (Welch and Black, 2010; Carter, 2017).

Medicalization and over diagnosis place great strain on individuals who are assigned such a diagnosis and suffer the resultant stress and perhaps even receive treatments of questionable benefit. Related economic costs rise and the indelible coding of medicalization or over diagnosis in the electronic medical record/patient chart or population vital statistics remain difficult to adjust or erase afterwards. Disease should be defined meaningfully, the risks and benefits of labels considered in the face of advancing science (Tikkinen et al., 2012). Proof of disease and amelioration or worsening of condition must be recorded. After being assigned, a disease label is static in the patient record and fixated in the patient's mind whether beneficial or not (Illich, 1976; van Dijk et al., 2016). Thus any change in disease definition must be considered carefully particularly when addressing a New Taxonomy. Modification of disease definition must not present unclear definitional boundaries or movable lines and thresholds that are readily transgressed.

The British Medical Journal has published an extensive series of articles on medicalization and related problems. Moynihan, one of the leading authors, writes of elevated cholesterol, hypertension, hyperactivity disorder, abdominal hysterectomy, and similar medical topics captured by medicalization and over diagnosis (Moynihan et al., 2002, 2014; Moynihan and Doust, 2012). To address medicalization and over diagnosis, Pathirana and Moynihan recommend more testing, more granular evaluative diagnostics and deeper, stricter requirements to warrant a diagnosis. They suggest that a more precise disease definition is achieved through additional supporting laboratory and radiographic information. However, medicine is long recognized as an art where imprecision characterizes areas where firm science might be lacking or yet to be proven associated with symptoms. Conversely, Black writes that the ability to detect finer laboratory or radiographic abnormalities tends to increase the incidence of disease diagnosis without always providing meaningful difference (Black, 1998). A further problem of relying on deeper, more refined laboratory and radiographic testing is that of assigning the potential for disease to occur in circumstances where it is not apparent and may never appear; uncertainty rules until true disease is detected (Pathirana et al., 2017). In summary, more testing, more granular evaluative diagnostics as recommended by Pathirana and Moynihan can actually exacerbate the problem of medicalization unless an appropriate scientific framework and relevant models exist; granularity linked to lines and static threshold boundaries does not solve the problem.

Hofmann explains that health care professionals themselves may not agree on the description of a particular disease. When questioned, professionals were able to list more typical and less typical disease expression. However, for actual coding purposes, Hofmann believes that there should be a means to correct any discrepancy, rather than have codes that are assigned and then perpetuated without explaining nuance (Hofmann, 2017). Not only is the ICD system poorly designed to express the nuance of disease and clinical change but the syntax cannot express the complex nature of medicine as it is so influenced by the tree-like hierarchy related to its underlying origins of botany classification. The problem of the reproducibility of medical information across large health care system datasets is another example of the problems with existing disease classification. Review of medical records suggests that targets for consistency when mining different datasets to reach a conclusion do not lead to reproducible results (Hucklenbroich, 1988). To help solve this problem of vast but inconsistent disease datasets across the population, the National Institutes of Health has recommended the establishment of a Data Commons, a large dataset pooling available information for investigation and comparison to replace the many individual, inconsistent datasets that span the many separate health care systems in existence today (Kannan et al., 2016). But without a proper framework structure for such a vast dataset, there is no assurance that the Data Commons will be successful. It may only perpetuate the underlying problems that exist in disease definition and record keeping and maintain the static nature of ICD and the erratic nature of disease definition (Manrai et al., 2016).

Since the genome has been described, there are few guiding principles to determine which genomic information related to disease is truly significant, vs. that information which may be of great interest but awaits verification. In the attempt to find genomic patterns and factors of disease, the path from genotype to phenotype is tenuous, neither direct nor certain and a Data Commons is not an assured solution (Weiskopf and Weng, 2013; MacArthur et al., 2014).

The difficulties that physicians face in the practice of medicine are succinctly summarized by Correia who writes that physicians must apply abstract principles of disease and discovery to concrete situations. “Specific ‘truths’ are supported by the discipline of medicine, but the application of such truths in the face of a changing patient condition may be only partly responsible for intervention and actual healing” (Correia, 2017).

## The philosophy of medicine—static labels, linear boundaries, and thresholds

Classification in medicine is recognized for its difficulty, for disagreement about instantiation, universals and ontology. Nevertheless, there remains the very real need for a practical, widely-used system and ICD and related classifications represent compromise and a valuable means to organize vital statistics for national and economic needs despite problems with bedside clinical utility or the gap between bench research and medical record disease assignment. Disease definition is therefore considered carefully in a world where the previously mentioned problems of medicalization and over diagnosis remain problematic (Brochhausen, 2017). The definition of disease is a “central” issue for medical philosophy (McWhinney, 1987). One looks to medical philosophy, therefore, for guidance when considering a New Taxonomy of disease and the incorporation of massive amounts of leading edge scientific information.

Recalling the foundational view of disease described by Sydenham, Hucklenbroich writes that “disease entity” is the key theoretical concept of medicine. He emphasizes that disease is like any entity, such as oxygen, an entity bounded either physically or metaphorically (Hucklenbroich, 2014). Thus, more than a label, more than symptoms, disease has characteristics and borders which can be clearly defined, lines which can be drawn.

This view is supported by Matthewson who believes that normal and pathological characteristics have objective distinction. A pathological state represents a condition where something has gone wrong. The concept of “wrong” is associated with physiologic response and change; the result is a disease that causes harm to a patient. The harm can be identified and assigned a label of disease. Harm is the distinctive feature (Matthewson and Griffiths, 2017).

Nordenfelt adds that diseases are more than clusters of symptoms and organ system related physiologic effects and measurements. Disease represents complexity and variability. This is how a physician interprets disease and communicates with a patient; disease is more than a label. The knowledge of a physician is contained in the use of terms and diagnoses from years of experience in following the course of patient disease expression (Nordenfelt, 2013).

The difficulties of lines and boundaries in disease definition are addressed by Doust and Rogers. Doust doesn't take issue with disease as entity but concentrates on the inadequacy when using the arbitrary, and movable, nature of boundaries (Doust et al., 2017). This view relates especially to the comparison of physiological function in response to disease, where alteration of function becomes arbitrary and true comparative patient groups nearly impossible to determine. Disagreements inevitably arise over where lines should be drawn and boundaries assigned, so that reduced physiologic function alone becomes a poor reflection of disease. Rogers discusses the problems with drawing lines to define disease when new technology offers additional measures (laboratory tests or radiographic studies) which might or might not support a particular diagnosis (Rogers and Walker, 2017). Patterns of disease may change over time; the question of degree of diagnosis arises as does certainty or severity. In Rogers's view, more tests and finer definitions do not achieve diagnostic accuracy or prevent medicalization or over diagnosis.

Lemoine concentrates on disease as explanation, to explain a patient's problem and explore risk factors. Instead of making a judgment when rendering a disease diagnosis, a physician presents an explanation. Lemoine links the implied causation that accompanies this explanation through a disease label to a patient's condition (Lemoine, 2013). This argument bears heavily on the investigation of genotype when it is related to disease phenotype expression; one must be certain that a patient genotype actually causes a subsequent disease phenotype rather than merely being present at the same time and thus of uncertain causative responsibility. Giroux emphasizes this causal inference of explanatory disease and questions how determinant genetic risk factors may or may not be (Giroux, 2016).

If one is going to formulate a New Taxonomy of disease, and it is meant to document a path from genotype to disease phenotype, there must be evidence of explanatory and causative features. This means more than the mere coincidence of factors. If explanation and causation are not expressed, one faces the problems of coincidence and ambiguity and the reality that perhaps only another vague line or boundary for disease has been created (Mekios, 2017). Lines, thresholds, and definitional boundaries have proven inadequate to add the wealth of genomic and molecular information to the understanding of disease except in those profoundly important cases where individual, select, genomic sites can be associated directly with disease expression. A more comprehensive explanatory framework of disease is needed, moving far beyond much current philosophical discussion that recognizes the problems of lines and boundaries but does not offer a solution.

## Network physiology—bounded components restricted largely to a single level of an organism

Physiology represents the key component architecture of the ICD and related disease classifications and is a proper place to revisit disease definition and taxonomy. Physiology traditionally relies on mechanistic models, achieved through reductionist process, models for explanation and discovery of function, whether at the cellular, functional tissue unit or organ system level. The reductionist mechanistic language of physiology facilitates learning and execution by healthcare practitioners and communication with patients for understanding. Physiology when expressed as reduced components and reassembled processes commonly functions well for linear, sequential explanation, and investigation. Common, technical word names for disease are supported with knowledge as to how the body responds.

Network Science is the fundamental method to study vast numbers of elements on a pre-determined level that compose a system. Physiology adopted the principles of Network Science, especially that studied by Barabasi. Barabasi examines systems of innumerable components and increasing complexity, whether communication sites on the internet, generators, and transmission lines for energy, or genes in the genome (Barabasi et al., 2011). He has developed network principles that describe relations and behaviors of elements that reside in a common spatial level, or layer, of a system. Individual components are called Nodes and graph theoretic charts are used to display relations with other Nodes through Edges, lines drawn to represent functional relation. Nodes are found to be “scale-free,” that is, all Nodes do not have an equal number of Edges (associations with other Nodes); rather, a more limited number of Nodes, called HUBS, have a great number of Edge relations while Nodes on the periphery tend to have few Edge relations (Barabasi, 2016). Networks commonly can be scaled to include enormous numbers of elements; because the elements are similar and reside on the same level, computation may be scaled exponentially.

Applying the tools of Barabasi and Network Science to the genome and inner-workings of the cell, Ivanov charts genome and molecular activity within the cell and formulates models of physiology. The discipline is called Network Physiology; disease is assumed to result from disease-causing Nodes or clusters of such Nodes at the genome level (Barabasi et al., 2011; Ivanov et al., 2016). However, Network Physiology relies on Nodes of one level within the organism—in this case the molecular level of the cell—and does not readily or necessarily include information from other biological spatial levels such as the cellular, functional tissue units, and organ levels. Network Science, and consequently Network Physiology, describes elements linked on a common level for description. Thus, without communicating channels between and among varying levels, and without recognizing that descriptions on one level (perhaps the genome) may have little direct application to other levels (perhaps functional tissue unit or complete organ system), Network Physiology remains quite limited in explicative power.

Through examination of the network components of the genome at the intra-cellular level, Ivanov views each organ system in the body as separate and subject to its own regulatory mechanisms; but beyond the network structure at the molecular and genomic level it becomes difficult to model entire cells or organ systems as communication channels resist description and physical constraint is poorly understood (Ivanov et al., 2016). Bartsch, working with Ivanov, adds the further problem to model distinct physiologic states in the face of the continuous nature of physiologic information (Bashan et al., 2012; Bartsch et al., 2015).

Addressing the Network Physiology model, Bartsch remarks that one cannot include inherent biologic oscillations and network communication channels, which transmit information from genome to cell to organ and back (Bartsch et al., 2015). Process oscillations and circadian rhythms are natural and vital to organism function. Communication signals are often weak and asynchronous; timing is not included in Network Physiology as the challenge is the nanosecond time scale of the genome vs. the minutes of the organ system and the years of the organism. Change at one spatial level in biology is not scalable to another using Network Physiology.

The investigative method of Network Physiology is reduction to the smallest component, the molecule and genome, but it awaits the theoretic reassembly through cells to functional units. It has been a major step forward in formulating our view of the genome, but it remains distant from actual mapping through to higher spatial levels of an organism; all the components within the cell have not yet been realized and the communicating channels and regulatory components between biological levels have not been clearly depicted. In addition, the linear, sequential mechanistic process of Network Physiology produces models that do not fit the varying time scale of components. The time scales of an organism—nanosecond to minute to hour to year—are vital for future research (Bechtel, 2017b).

Physiology is increasingly seen as complex, non-linear, and non-sequential; states and communication channels are intermittent while physiologic messaging is asynchronous. Network Physiology is challenged by the fact that each organ system maintains its own time scale for reference and re-composition; variation in function is subject to constraints of communication (Goldberger et al., 2000; Bechtel, 2017c). These difficulties are emphasized by Moorman and Ivanov in their discussion of the early detection of sepsis (widespread infection through the body resulting in organ system dysfunction) (Moorman et al., 2016). They, as do other researchers, refer to physicists who long ago learned that integrated functions at the system level cannot be simply expressed as the sum of individual subsystems and their behaviors (Morrison and Newell, 2012; Green, 2013; Pantziarka, 2016; Batterman, 2017; Goulev et al., 2017; Nagy et al., 2017).

The problem of a reductionist static biologic model, like Network Physiology, is explored by Liu who analyzes the major components of dynamic networks of physiologic organ interactions. He notes that the behavior of one system affects the dynamics of other systems; thus, dynamic system theory may be more appropriate for the study of physiology but such models remain a quite distant target (Liu et al., 2015). For example, Sherman describes cell membrane excitability that can change with time resulting in different cellular behavior and a consequent variety of responses to stimuli (Sherman, 2011). It is known that much in biology is non-linear, that sequential linear and simplified description is not entirely accurate, although such description remains useful for medical bedside models and bench research study.

Qu summarizes this argument noting that biological systems are multi-scale, functioning, and communicating across measures ranging from the nanometer of molecular dimension to the meter of a living organism confined by temporal constraints (Qu et al., 2011). Dynamic behaviors across these ranges of space and time are complex and resist facile description.

Network Physiology is important in the consideration of disease definition and a New Taxonomy; however so many multi-level problems of time and space remain as disease progresses that one must search further. The need for Scalable Design enters the discussion as it is really the limitation of Network Physiology to a level of the organism and the consequent inability to model change over time that restrict its application to a New Taxonomy. Disease is not confined to one part of the genome, individual cells, or even identifiable functional tissue units, or to one moment in time. Disease exists throughout the organism whether through messenger signals, inflammatory response or regulatory cascades. To be informative, a New Taxonomy must contain these scalable characteristics; if these characteristics are not included, one is left with separate genome sites or molecular identities. The complex scalable interaction, the interface of organism and causative agents, is missing.

## Network medicine—bounded disease models, single level, confined in space and time

Much of contemporary medicine relies on the efforts of physicians in the nineteenth century who linked bedside diagnosis with disease entities, and the subsequent world of laboratory and radiographic discovery. Loscalzo suggests a new paradigm of disease, Network Medicine (Walker, 1990). This paradigm is meant to bridge the gap between genotype discovery and disease phenotype expression, as similarly suggested by the NAS and the recommendation for a New Taxonomy of Disease; it would describe disease type more specifically for each patient through genome identification and organized supporting science (Desmond-Hellman et al., 2011). Network Medicine applies the approach of Network Physiology to disease discovery, matching genome findings through Network Science with the expression of disease phenotype. Central to Loscalzo's paradigm is the belief that disease is the result of a mutation found on the genome and that disease itself is an entity. Locate the mutation and a path to disease phenotype can be traced (Chan and Loscalzo, 2012; Silverman and Loscalzo, 2012).

The method of discovery in Network Medicine, much like Network Physiology, relies on the historical Cartesian process of reduction to smallest component, in this case the gene, and then reassembly into a model of disease phenotype through association with the electronic medical record (Walker, 1990). There have been many notable successes through this method, but progress has slowed and become intermittent (Bar-Yam, 2004). Approximately ten percent of genes have been associated with disease and not necessarily in a consistent manner with identical disease phenotype; disease associated genes tend to be on the periphery, which means few Edges and relations with other Nodes. HUBS are more central and well-connected through Edges and consequently not commonly associated with disease (Korcsmaros et al., 2017).

Other researchers have pursued the concept of the diseaseome, extending the concept of Network Medicine to include clusters of genes that together are expected to explain disease rather than concentrating on individual gene mutation (Caldera et al., 2017). That is, disease phenotypic expression would be explained by several genome sites functioning together, probably located in close proximity (Menche et al., 2015). But such genome sites associated clearly with disease are the minority and tend to be peripheral; the complexity of computation is extraordinary and the ability to prove that clusters of locally associated genes lead to disease remains elusive. Writing of a functional disease module of related genes, Goh pursues this model of a human disease network determined by clusters of functioning genome elements. This work faces the challenge of tracking disease genotype transmission beyond the cell, as there remains an absence of defined communication channels between spatial biological levels. Pathways of organism inflammatory response or changes in coagulation cascade associated with gene clusters remain unidentified (Goh et al., 2007). In summary, Network Medicine as a model for disease does not scale. It tracks a direct path between genotype and phenotype, omitting the actual varied transmission channels, which describe disease variation and exacerbation. It cannot account for non-linear processes and emergent properties.

The question of the nature of the gene itself further confounds a reductionist focus on genotype to fully explain disease phenotype. The gene is not necessarily a fixed, identifiable quantity and may have varying expression over time. Genes may associate with other genome sites that can modify expression or effect (Li and Agarway, 2009). Research suggests that the gene can change, that a gene associated with congestive heart failure can be altered as a result of the disease process, making computation, and consistent phenotype assignment problematic. A gene may have inconsistent compositional elements and lack predictable qualities (Portin, 2002; Portin and Wilkins, 2017). If such variability in expression or association is accurate, Network Medicine should explore a malleable gene concept and the resulting uncertain association of gene site with specific disease rather than maintaining the belief that disease is an entity and the result of gene mutation. More work will need to be done before Network Medicine can claim to be the definitive means to link genotype and disease phenotype (Yang et al., 2016).

## Case studies, the limits of lines, boundaries and networks

Disease description and classification work well for population vital statistics, health system medical maintenance, and economic profiles. Medicine functions at the bedside with names, mechanistic reductionist models, and a computer interface which essentially records laboratory, x-ray, and staff input data. But the wealth of forthcoming genomic, molecular (OMIC), and biomedical information overwhelms current knowledge bases (Campos et al., 2013; Weinberg, 2014; Shah and Sureshkumar, 2015; Stephens et al., 2015). The following brief case scenarios highlight further concerns about disease definition and the need for a more robust framework.

### Pre-diabetes

The condition of an intermittently elevated blood glucose level, not sufficient to warrant a diagnosis of diabetes, in an individual not certain to develop the full syndrome of diabetes later in life. There is no accepted genomic signature to provide clinical guidance. It is not known with certainty which patients will proceed from pre-diabetes to later disease. Repeat examination and attention to diet and exercise over many years are advised. However, pre-diabetes is an amorphous concept, which lacks markers and models of evolution. The pathways the organism follows, whether at the micro vascular, the metabolic, the nutritional regulatory, or the inflammatory levels, require discovery to chart the pathway that the entire organism follows over years prior to exhibiting diabetes. Once diabetes does appear, the complex interplay throughout the organism needs description and tracking to document the influence of cells and organ systems on each other.

### Huntington's chorea (HD)

A progressive, devastating neurological disease characterized by worsening choreiform movements (involuntary) worsening over many years. This is an inherited condition: those individuals with more than 40 repeating segments on the Huntington Gene suffer debilitating symptoms during life; fewer than 36 repeating gene segments may carry the inheritance of Huntington's but individuals do not suffer symptoms; those individuals with 36–39 repeating gene segments face an agonizing lifetime without a means to predict whether or not the choreiform movements will eventually occur. One can only look retrospectively. In contrast, to understand HD specifically in those individuals with only 36–39 segments, science needs understanding of neurological pathways and excitable muscular units and their function over the years prior to the appearance of actual choreiform movements. The progressive deterioration after HD actually appears also needs to be explored.

### Torso trauma and severe coagulopathy

Severe, massive torso trauma to a previously healthy individual who promptly develops a poorly understood inability of blood to clot properly (coagulopathy). The common coagulation pathways and laboratory studies have not offered a convincing explanation for the coagulopathy and treatment thus remains problematic. In conjunction with coagulopathy, many systems in the body collapse simultaneously and survival becomes uncertain. No signature or cluster of pre-disposing factors has been determined for this devastating acute reaction to trauma. These changes may be overwhelming and unpredictable; they may not recur as it is the complex interaction of extent of trauma, organism preparedness, molecular, and cellular response and the balance of fortune at any given time.

Each of these cases presents extreme highlights to demonstrate the need for viewing disease (pre-diabetes, HD 36-39 gene segments but without symptoms, torso trauma and coagulopathy) as a process. Changes occur progressively over time, or massively and acutely, and involve multiple levels of the organism; in the case of pre-diabetes and Huntington's with 36–39 segments multiple levels of the organism become progressively involved, but there is no effective means to track or predict accurately. In the case of the torso trauma coagulopathy, a devastating, acute systemic process begins immediately. These cases represent problems of process without clear lines and boundaries; they are not accounted for meaningfully through disease classification and are not described by Network Physiology and related medical models. Change occurs across spatial and temporal scales in a discontinuous manner that suggests complex behavior and emergent outcomes.

## Systems biology and systems medicine—scalable, adaptable, flexible definitions across time and space

There is a disconnect between what is accepted as explanation—how we model and describe—and how the world really works. Our traditional models of biology and how the human body behaves function well enough in the clinic, the emergency room, the insurance billing office and the public health service (Altaf-Ul-Amin et al., 2014). These descriptions follow reductionist mechanistic explanatory principles by reducing to the smallest component and then reassembling for clarity and utility (Mobus and Kalton, 2015). Readily understood lines and thresholds are used; definitional boundaries are recorded. However, scientific advance, the explosion in genome related research and the wave of latest monitoring technology (micro-sensors and OMICS) compel re-examination of disease classification and biologic models (O'Malley et al., 2007; Saetzler et al., 2011). When science adds knowledge that does not fit readily into the more traditional patterns, the basic conceptions should be re-explored.

Systems Biology and Systems Medicine are the means to take on this challenge, to improve biologic classification far beyond the mechanistic reduction to smallest components with subsequent re-composition (Breitling, 2010; Rajapakse et al., 2012; Brigandt et al., 2016; Bechtel, 2017a). A systems approach is not antithetical to the traditional reductionist to smallest components, but should be seen as working in tandem, with different expectations for each point of view (Mossio et al., 2013; Pezzulo and Levin, 2016). Systems theory includes overlying organizing principles, which through their existence exert organizational forces on lower system elements (Ramoni et al., 2017; Rivas et al., 2017). The lower systems are composed of subsystems, in multiple spatial layers, which communicate through channels and over temporal periods as varied as nanoseconds and years. Such is the human.

Systems Biology derives inspiration from Bertalanffy who realized that reduction to smallest components and assembly into mechanistic models was helpful but did not explain biology completely; an overarching hierarchy of influence by the structure itself had to be included in biologic explanation (Navlakha and Bar-Joseph, 2015). Subsystems are constrained by the structure of the entire organism, called constitutive constraint by Craver, and exist on multiple spatial scale levels within the organism (genome, cell) and function across vastly different time scales (nanoseconds to years) (Craver and Bechtel, 2007). There is a tyranny of scales that characterizes the physical properties of collective elements, the physical constraints dictated by composition itself at the cellular, functional tissue and organ levels (Goroshowski et al., 2011; Ellis, 2012; Green and Batterman, 2017). The subsystems communicate through asynchronous channels and are managed by distributed autonomous subsystem control. This is more closely related to a distributed model and dynamic systems design than it is to individual gene site mutations, the foundation for genomic research and related Network Medicine (Goroshowski et al., 2011). The biological subsystem levels of the organism communicate, messaging is intersecting and competitive; regulation does not occur through linear process but rather through competitive molecular binding sites and cascading inflammatory, coagulation, and organ system messaging (Havel, 2007; Westerhoff et al., 2009; Kaiser and Krickel, 2017).

Wolkenhauer writes of the transition from Systems Biology to Systems Medicine. Current efforts must embrace the emergent properties of disease through models consistent with non-linear processes (Wolkenhauer et al., 2013). This mirrors the conclusion of Moorman and Ivanov that Network Medicine alone is unable to create a model of sepsis (overwhelming systemic body infection and response) from reduced components (Moorman et al., 2016). Pneumonia is much more than a finding on chest-ray or a cluster of symptoms. Loscalzo's Network Medicine to link genotype and phenotype seeks a more specific understanding of disease; but the requirement of more specific disease definition remains unmet through this approach (Fernandez et al., 2013; Brown et al., 2016). The complexity of biology, the non-linear mathematical and computational models of an asynchronous dynamic nature, must be incorporated into Loscalzo's major Network contribution. Multiple biological levels and their related separate networks must be included along with the many communication channels determined (Shin and Brodsky, 2015; Garland, 2017). The obstacle of the tyranny of scales that lies along the path of models that unite findings on one level (genome, cell, functional tissue unit, and organ) to another must be recognized.

Systems Medicine relies on mathematical and computational models for these next stages as a purely reductionist methodology is overwhelmed by the number of factors and crossing pathways. Ideker writes of this problem as Systems Design moves forward: that fluctuating network, components, and structures may be required in our future models of biology (Ideker and Krogan, 2012; Furlong, 2013). How else to explain the prolonged path of multiple organ systems as an individual subtly slides from pre-diabetes to diabetes or the seemingly random devastation that strikes an anxious patient with the debilitating choreiform movements of Huntington's Disease or the path from infection to sepsis?

Of equal importance is Wolkenhauer's emphasis on time elements; very much like Ivanov and Bartsch, he explores the need for Systems Medicine to recognize the time scales of biologic processes, for the differences that result from weak signals and the competition for molecular receptor response (Wolkenhauer et al., 2013). Bechtel, like Noble's work on the heart, emphasizes that circadian rhythms and cellular process oscillations cannot be modeled from the bottom up. He describes different gene mutations that result in similar phenotypes, suggesting temporal and competitive factors (Bechtel, 2017a). Green refers to cancer as a dynamic attractor state; the question is raised whether cancer is an emergent property within a hierarchy of organism constraints (Green et al., 2017). This model may ultimately be applied more widely and explain why some patients develop respiratory symptoms while others do not when influenza races through a city (Feinerman and Korman, 2013). Through this description of biologic system theory, lines, and boundaries expand to take on the characteristics of state space described by Ereshevsky, a yet to be determined model for disease (Ereshefsky, 2009). The problems with medicalization result mostly from lines and boundaries which are applied to describe “something” that really spans time and space through multiple dimensions rather than residing neatly between clean, precise thresholds (Ching et al., 2017). Views such as that of Kovac, that biological information is more than genome elements, add another dimension (Kovac, 2007; Wertheim, 2015; Suderman et al., 2017). Thermodynamic laws come into play and help determine which signals are translated to information, to process and change, vs. those signals which are not (Yan and Charles, 2017). Gershenson summarizes information in biology as a process which depends on where, and when, it is received; timing, asynchrony and competition are all important, a topic related to Bechtel's chronobiology (Gershenson, 2012; Gershenson and Fernandez, 2012).

## Systems medicine and disease

Systems Biology addresses the gap between current science and traditional understanding and translates to Systems Medicine. The definition of disease is the key to this transition. A static nomenclature, such as ICD coding labels and the view that disease is an entity to be described in entirety (a view that harkens back to Sydenham in the seventeenth century), although useful in many disciplines, are not consistent with systems analysis. Currently a Systems definition of disease is missing in this discussion (Ramoni et al., 2017; Rivas et al., 2017). Without such description at the interface of the organism and disease, difficulty persists matching Systems Medicine with a largely static, historically founded concept: disease described as an entity confined by lines and boundaries. In reality, Disease participates within a system, at the interface of causative factors and organism response, interacting through multiple spatial levels and measures of time (Hanselmann and Welter, 2016). Dupre refers to this as a process and the interplay of subsystems (Dupre, 2006; Dupre and O'Malley, 2007; Dupre and Guttinger, 2016).

The organizing principles, the constraints imposed by the organism itself on the lower levels and component parts, have yet to be clearly determined and need to be included. Systems Biology describes a dynamic, adaptive, complex organism with decentralized, autonomous control, and emergent properties. Disease exists in synergy with this organization, not separate, not divided by lines and boundaries, but scalable and participating in malleable networks at varying levels of messenger interception (Hetz and Glimcher, 2011; Cvijovic et al., 2014; Green et al., 2017; Hill et al., 2017).

## Interface of organism/disease—the need for scalability

To complete the transition from Systems Biology to Systems Medicine and address a New Taxonomy of Disease, an additional feature is required: the interface of organism and Disease across space and time. Systems Medicine includes all systems and subsystems as well as response and communication channels. The multi-level spatial composition of the organism has been described (genome, cell, functional tissue unit, organ system), but the interface with Disease has a further feature—the passage of time. Organism response depends on the age of each unit with the understanding that parts of the organism may manifest the effects of aging, especially related to change with Disease, at different rates and to varying degrees. That is, subsystems of the organism do not age all at the same rate, and physiologic expression of the aging process may proceed intermittently (diabetes may have a more profound effect on retina and kidney function initially, Huntington's Disease may have devastating effects with age).

The varying rates of aging compound the difficulty to determine the rate of change at the organism/disease interface making scalable description problematic. Systems Medicine addresses the time element of Disease both with the knowledge of change related to aging as well as the characterization of the organism/disease interface over extended periods. Time is addressed through chronicity and through the constraints of aging. The process is not linear. Time may even be extremely compressed as in the case of massive torso trauma and acute coagulopathic response but the interface description remains.

Nevertheless, a New Taxonomy of Disease must be scalable beyond networks and beyond ordinary static boundary conditions. Disease itself is dynamic; it changes expression depending on interacting spatial level communication, inflammatory response and the temporal involvement of subsystems. To be useful, the New Taxonomy will include the interface of organism/disease and offer the scalable characteristics of Disease expression not included in names, ICD codes, networks or static boundaries.

The difficulty of scalable metrics in biology has been examined. Higgins writes that in a complex system such as human biology, a single formalism cannot account for all of the properties (Higgins, 2002). This means that many different approaches might be included in a larger framework for Disease Taxonomy depending on specific goals and utility. Nousala notes that scalability is fundamental to navigate the system levels of complex systems as so many conflicting variables may be at work (Nousala, 2013). Jogalekar emphasizes new scalability metrics for distributed networks, much like Systems Medicine Disease, particularly in the presence of increasing numbers of variables and multi-dimensionality (Jogalekar, 2000). West has described the scalable mathematics for many observations in biology, whether the size of an organism related to metabolism or the branching nature of arterial channels and microvasculature, power laws offer scalable explanation (West, 2014). However, scalability must be useful to be incorporated into a Systems analysis and a scalable design for Disease remains largely descriptive. The outstanding problem with organism/disease interface is the difficulty to offer a scalable portrait of interaction. Disease exists on multiple spatial levels and time periods, asynchronously. For true understanding the principles which govern the interface, the ability of Disease to interact simultaneously at multiple points on the interface, need discovery. Disease is scalable from the molecular to the entire organism, acutely and over time. A New Taxonomy must make use of innovative models and analytics. The inflammatory and communication channels of the organism/disease interface, whether modeled on OMIC patterns and rates of change or cellular models, require confirmation.

## OMIC patterns and rates of change—scalability toward a new taxonomy

Systems Medicine Disease must link the subsystems of the organism and include features of both organism and disease at their Interface. The multi-level spatial constraints of biology and feature change that occur over time characterize these processes (Furness, 2017). Time expressed as aging has an effect on disease and organism response (Zierer et al., 2016) that a supplemental New Taxonomy must inform in conjunction with current classification and coding methods. The subtle system-wide molecular pathways that drive pre-diabetes to an elevated glucose and insulin injections or the tragic late-onset of Huntington's choreiform seizures require illumination long before disease occurrence. Chronic disease benefits when identified in the absence of symptoms or physiologic change.

OMICS and their vast disparate datasets comprise molecular components of genome (genomics), the products of genome code transmission (transcriptomics), cellular proteins, and communication elements (proteomics) and molecules reduced through metabolism (metabolomics). The study of epigenomics demonstrates that while a gene may be static once characterized, its effect may be varied in combination with other genes or multiple communication channels (Pineda et al., 2015). Unlike genomics, whose foundation remains stationary, OMICS embraces change across the spatial levels of the organism (from genome and cell to organ systems) and, through repeated analysis and measured time, can describe rates of change and multiple patterns of participation. OMICS can illuminate the information transfer across Networks themselves confined to one spatial level of the organism. Current OMICS datasets are overwhelming, not organized for systematic study and application; innovative computation techniques are required to orient the datasets to best definition through streamlining (Hasin et al., 2017).

The use of OMICS to focus association with biologic processes and disease phenotype lie in the future along paths which most certainly will not be linear. Two goals arise—(1) delineate the Interface of organism/disease to recognize response to disease and change in offending etiology as it occurs and (2) recognize change at the organism/disease Interface of chronic disease as early as possible to enable true prediction (Acharjee et al., 2016). OMICS study can be repeated as often as necessary with the proper technology; significant computation is required to sift the molecular results and search for association, whether related to specified biologic processes or to subsequent disease phenotype.

Clearly, the current status of OMICS datasets does not yet permit the analysis of vast disparate datasets to determine all of the communication channels and inflammatory response pathways (Buescher and Driggers, 2016; D'Argenio, 2018). However, early successes demonstrate that with the proper approach the Interface of organism/disease can be understood and add a systems point of view to Disease (Sun and Hu, 2016; Argelaguet et al., 2018). Preliminary examples include the study of metabolic products that track energy processes associated with heart disease and functional deterioration, the OMICS changes that drive the development of scarring in pulmonary fibrosis and the molecular proteomics that signal the deterioration from septic shock (Kan et al., 2017; Bakker et al., 2018; Cambiaghi et al., 2018). These examples confirm the grander possibilities of OMICS at the Interface: to track molecular communication or response throughout the organism and at the organism/disease Interface as boundary conditions of disease expand or contract, whether influenced by age or confounding factors of co-morbid disease. OMICS offers the distinct scalable advantage to delineate the biologic information channels, no longer confined to a single genome site and linear, mechanistic progression or to a moment of observation and thereafter static classification (Ohashi et al., 2015; Huang et al., 2017; Tsuyuzaki and Nikaido, 2018).

## Conclusion

A New Taxonomy of disease is requested to supplement the existing ICD and related classifications. It becomes clear that current disease description, whether historical names or ICD code labels, lacks many key features. Pressure builds for reconsideration. An inability to manage the forthcoming massive amounts of leading edge scientific discovery and the static nature of contemporary methods must be addressed. The addition of new names for disease along with associated lines and boundary thresholds is not helpful. Increasing alphanumeric ICD codes as science advances has reached its limit.

Fresh consideration of disease and a New Taxonomy is required which will incorporate concepts of Systems Medicine (Kaplan et al., 2013), recognize Systems Medicine Disease and include the organism/disease interface.

A key feature of Systems Biology is the description of the many subsystems of the organism, the dynamic interplay of complex, adaptive subsystems quite removed from reductionist mechanism. Systems Medicine includes Disease in a systems point of view. In addition to the common nomenclature and clinical vocabulary (disease names and ICD codes), the nature of Systems must be added to the classification of Disease and the interface of organism/disease addressed. This interface of the causative agent(s) and its impact on and response of the organism is expressed across varied spatial levels and individual components. Disease is progressive through time, whether toward improvement and “cure” or the misfortune of further deterioration and the subsequent enlisting of organ system response and related insufficiency. Deterioration transmitted through common communication channels, yet to be finely delineated, suggests cascading inflammatory pathways that channel the organism's response to disease.

Systems Medicine Disease views disease at the interface with the organism; etiologic factors and biologic response refine classification across multiple spatial levels, the passage of time, and the ravages of aging.

## Author contributions

RB oversaw the topic discussion and organization of the manuscript. RG added the discussion of medicalization and relation to physiology investigation as well as case study. JB considered the problems of genomic, metabolomics discovery, the difficulties translating this knowledge to medical curriculum, and the gap that remains with disease definition.

### Conflict of interest statement

The authors declare that the research was conducted in the absence of any commercial or financial relationships that could be construed as a potential conflict of interest.

## Footnotes

^1^International Classification of Diseases, Tenth Revision, Clinical Modification (ICD-10-CM), 2018 https://www.cdc.gov/nchs/icd/icd10cm.htm (Accessed March 4, 2018).
